# Axial Stress Measurement of Small Diameter Aluminum Alloy Tubes Using a Conformal Oblique Incidence-Guided Wave Probe

**DOI:** 10.3390/s26206450

**Published:** 2026-10-12

**Authors:** Shuangxu Yang, Chunguang Xu, Yongjiang Ma, Wenjun Zhang

**Affiliations:** 1 School of Mechanical Engineering, Beijing Institute of Technology, Beijing 100081, China; 2 Ningbo Branch of China Academy of Ordnance Science, Ningbo 315103, China

**Keywords:** ultrasonic-guided waves, axial stress measurement, L(0,2) mode, conformal oblique incidence probe

## Abstract

Quantitative evaluation of axial stress in small diameter thin-walled aircraft tubing is important for characterizing its mechanical state during assembly and service. However, restricted circumferential access necessitates a compact local excitation configuration, for which geometric mismatch between conventional planar acoustic sources and curved tube surfaces can lead to circumferentially nonuniform excitation and interfere with stable time of flight (TOF) estimation. This study proposes a reference-based axial stress measurement method using an L(0,2)-dominated guided wave response generated by a conformal oblique incidence probe. Based on acoustoelastic-guided wave analysis and transient response characteristics, 500 kHz was adopted as an appropriate operating frequency for the present sensing configuration. Three-dimensional finite element simulations showed that the conformal loading configuration produced a more regular circumferential wavefield distribution. Experimental comparisons showed that the amplitude of the strong subsequent response was approximately 91% of the target wave packet amplitude for the conventional planar probe but below 31% for the conformal probe, indicating greater time domain prominence of the target wave packet relative to subsequent responses and providing a more stable waveform basis for TOF estimation. Repeated calibration established an approximately linear relationship between TOF shift and axial tensile stress. Validation tests on tubes from the same batch, together with repeated measurements after probe reinstallation and recoupling, yielded an overall mean absolute deviation from the reference stress of 1.86 MPa and a mean absolute relative deviation of 3.17%. The results demonstrate the feasibility of reference based quantitative axial stress evaluation using an established zero-load reference state in small diameter thin-walled aluminum alloy tubes under restricted circumferential access.

## 1. Introduction

Small diameter thin-walled metal tubes are widely used in aircraft hydraulic, fuel, and pneumatic systems, with aluminum alloy tubes being among the most common types. Assembly and installation constraints can introduce stresses into aircraft tubing, and these stresses may contribute to fatigue cracking during service [1]. Such stresses can alter the mechanical state of the tubing system and may consequently affect its structural performance and service reliability. Among the stresses that may arise in installed tubing, the present study focuses on the axial stress component. Quantitative determination of the axial stress level is therefore necessary to characterize the actual mechanical state of the installed tube, rather than merely identifying the presence of stress. Accordingly, nondestructive quantitative stress measurement in small diameter thin-walled aircraft tubing is of practical engineering significance.

Existing stress measurement techniques include strain gauge measurement, X-ray diffraction, neutron diffraction, magnetoelastic methods, and ultrasonic methods. Strain gauge measurement generally requires gauges to be installed in advance or bonded locally to the structure, which can limit its applicability to in service tubing systems without preinstalled sensors. X-ray diffraction is mainly used for near surface stress measurement, whereas neutron diffraction can characterize stress within the bulk material. However, these techniques require complex equipment and involve relatively high operating costs. Their field applications are also restricted by limited accessibility and safety requirements [2,3]. Magnetoelastic methods are sensitive to the magnetic properties and magnetization conditions of the material and are therefore not directly applicable to nonferromagnetic aluminum alloy tubes [4]. Among these techniques, ultrasonic methods use the acoustoelastic effect to characterize the stress state through stress-induced changes in elastic wave velocity or time of flight (TOF). They offer portable instrumentation and good suitability for field measurements [5]. Conventional bulk wave methods have been applied to local stress measurement. However, the measurement region is generally localized and depends strongly on the acoustic path, incidence conditions, structural geometry, and transducer placement [6].

Compared with conventional bulk wave methods, ultrasonic-guided waves propagate in discrete modes constrained by structural boundaries and can travel relatively long distances along the tube wall. Their propagation characteristics are sensitive to the path averaged axial stress between the transmitting and receiving probes, making them suitable for axial stress measurement in tubular components. Gazis established the three-dimensional theory of elastic wave propagation in hollow circular cylinders and derived the corresponding dispersion characteristics of axisymmetric and nonaxisymmetric guided wave modes [7,8]. Silk and Bainton experimentally investigated ultrasonic-guided wave propagation in thin-walled metal tubing and examined the excitation characteristics of longitudinal modes including L(0,1) and L(0,2) [9]. These studies provide important theoretical and experimental foundations for guided wave propagation in tubular structures.

Guided wave-based stress measurement has also been investigated directly in tubular and pipe-like structures. Ma et al. applied ultrasonic-guided waves to stress measurement in aeroengine thin-walled tubing [10]. Liu et al. investigated stress measurement and influencing factors in aeroengine duct assemblies using guided waves [11]. Zhu et al. reported axial stress measurement in steel tubes using ultrasonic-guided waves [12]. Liu et al. further investigated the effect of axial stress on the propagation velocity of longitudinal guided waves in liquid-filled pipes and demonstrated the potential of these waves for monitoring stress levels in tubular structures [13]. Previous studies have shown that an approximately linear relationship may exist between guided wave velocity or TOF and axial stress within a certain loading range, while nonlinear responses may also occur depending on the structural configuration and loading conditions [14].

Cylindrical guided waves in tubular structures exhibit multimodal and dispersive characteristics. Different guided wave modes exhibit different displacement distributions, propagation velocities, dispersion characteristics, and stress sensitivities. Since the present study focuses on axial tensile stress measurement using an early arriving wave packet propagating along the tube axis, longitudinal modes were prioritized for further analysis. For guided wave stress measurement in tubular structures, stable excitation and reliable identification of the target mode are essential for accurate stress measurement. Circumferential excitation conditions can influence the generation of axisymmetric and nonaxisymmetric guided wave components. Zhang et al. investigated the excitation of axisymmetric and nonaxisymmetric guided waves by magnetostrictive transducers in hollow cylinders and showed that the circumferential loading length and loading distribution affect both the circumferential wavefield distribution and modal composition [15].

However, the space available for in situ inspection of small diameter aircraft tubing is limited. Although circumferentially distributed excitation methods, such as full circumferential piezoelectric transducers and ring arrays, facilitate the generation of relatively uniform axisymmetric longitudinal modes, their deployment requires access around a substantial portion of the tube circumference. In densely installed aircraft tubing, such access may be restricted by neighboring tubes, clamps, fittings, and surrounding structural components. A compact local oblique incidence probe configuration is therefore more compatible with the restricted circumferential access conditions considered in this study [16]. However, the effective loading produced by a planar piezoelectric element on a curved tube wall may reduce the local circumferential uniformity of the excitation, thereby increasing the contribution of nonaxisymmetric modal components and circumferential propagation components [17]. Because the stress-induced TOF variation is small, contamination of the target wave packet by nearby non target responses can alter its waveform shape and temporal boundaries, thereby increasing the uncertainty of correlation-based TOF estimation and subsequent quantitative stress evaluation. Therefore, improving the time domain prominence and waveform consistency of the stress sensitive target response is essential for reliable local guided wave stress measurement in small diameter thin-walled tubes.

Accordingly, this study develops a quantitative axial stress measurement method for small diameter thin-walled tubes under restricted circumferential access. The method integrates stress sensitive guided wave analysis, conformal local oblique incidence excitation, correlation-based TOF estimation, and experimental stress calibration and validation. The conformal probe is introduced to improve the time domain prominence and waveform consistency of the L(0,2)-dominated stress sensitive response, thereby supporting stable TOF estimation and quantitative axial stress evaluation. First, the dispersion characteristics and stress sensitivity of longitudinal modes in an aluminum alloy tube with an outer diameter of 18 mm and a wall thickness of 1 mm are analyzed under different axial stress levels. The L(0,2) mode within the candidate frequency band is first evaluated based on its group velocity, dispersion characteristics, and stress sensitivity, and the transient responses at candidate frequencies are subsequently compared to select 500 kHz as the operating frequency. Second, a pitch catch-guided wave probe pair based on conformal oblique incidence excitation is developed. Three-dimensional finite element analysis is used to compare the wavefield characteristics produced by the conventional planar piezoelectric oblique incidence probe and the proposed conformal probe, including their differences in circumferential wavefield distribution and more regular circumferential wavefield distribution characteristics. Representative experimental A scan responses obtained using the two probes are then compared to evaluate the time domain prominence and waveform consistency of the L(0,2)-dominated target response. Finally, an experimental system integrating axial loading and guided wave measurement is established. The TOF shift of the L(0,2)-dominated wave packet is extracted using normalized cross-correlation, and an experimental calibration relationship between TOF shift and axial stress is established and validated using specimens not involved in the calibration.

## 2. Acoustoelastic Guided Wave Propagation and Stress Mapping Under Axial Prestress

### 2.1. Stress-Dependent Effective Stiffness Under Axial Prestress

Axial prestress changes the effective elastic stiffness of an aluminum alloy tube and consequently alters the dispersion characteristics and propagation time of longitudinal guided waves. The tube considered in this study has an outer diameter of 18 mm and a wall thickness of 1 mm. A cylindrical coordinate system (*r*, *θ*, *z*) is adopted, with the z direction aligned with the tube axis.

The straight tube section between the transmitting and receiving probes is sufficiently far from the gripping regions and geometric transition zones. No additional pressure is applied to either the inner or outer surface of the tube. Under stable axial loading, bending effects within the propagation region can be neglected. Therefore, this region can be considered to be under a uniform uniaxial tensile state. The nominal axial stress is expressed as

(1) $$\sigma_{z}=\frac{F}{A_{0}}$$

where *F* is the axial tensile force and $A_{0}$ is the cross-sectional area of the tube in the undeformed state. A positive value of $\sigma_{z}$ denotes axial tensile stress.

Under the assumptions of small deformation and linear elasticity, the initial strain components corresponding to the uniaxial tensile state are expressed as

(2) $$\left\{\begin{array}{l} \varepsilon_{rr}^{0}=\varepsilon_{\theta \theta }^{0}=-\frac{\nu }{E}\sigma_{z}\\ \varepsilon_{zz}^{0}=\frac{\sigma_{z}}{E}\\ \gamma_{r\theta }^{0}=\gamma_{\theta z}^{0}=\gamma_{rz}^{0}=0 \end{array}\right.$$

where *E* is Young’s modulus and *ν* is Poisson’s ratio.

Ultrasonic-guided waves are treated as small amplitude dynamic disturbances superimposed on the static predeformed state. According to the incremental theory of elasticity for predeformed solids, the effective elastic stiffness under prestress can be expressed as [18,19]

(3) $$\begin{array}{c} C_{ijkh}^{\sigma }={c_{igkh}w}_{j,g}+c_{ijgh}w_{k,g}+c_{ijkg}w_{h,g}+c_{gjkh}w_{i,g}\\ +\left(1-w_{p,p}\right)c_{ijkh}+\tau_{ik}^{0}\delta_{jh}+c_{ijkhpq}\varepsilon_{pq}^{0} \end{array}$$

where $c_{ijkh}$ is the second order elastic constant tensor in the unstressed state, $c_{ijkhpq}$ is the third order elastic constant tensor, $w_{i,j}$ is the static displacement gradient, $\tau_{ik}^{0}$ is the initial stress tensor, $\varepsilon_{pq}^{0}$ is the initial strain tensor, and $\delta_{ij}$ is the Kronecker delta. Repeated indices follow the Einstein summation convention.

For the theoretical analysis, the aluminum alloy is approximated as a homogeneous isotropic Murnaghan elastic material [20]. The second order elastic constants are expressed in terms of the Lamé constants as

(4) $$\lambda =\frac{E\nu }{\left(1+\nu \right)\left(1-2\nu \right)},\;\mu =\frac{E}{2\left(1+\nu \right)}$$

The material parameters used in the analysis are the density $\rho_{0}=2730\;kg/m^{3}$, Young’s modulus *E* = 69.1 GPa, and Poisson’s ratio ν= 0.354. According to Equation (4), the corresponding Lamé constants are λ= 61.87 GPa and μ= 25.52 GPa. The Murnaghan third order elastic constants are taken as *l* = −201 GPa, *m* = −305 GPa, and *n* = −300 GPa [21]. In Voigt notation, the relationships between the third order elastic constants and the Murnaghan constants are given by

(5) $$\left\{\begin{array}{l} c_{111}=2l+4m,\;c_{112}=2l,\;c_{123}=2l-2m+n\\ c_{144}=m-\frac{n}{2},\;c_{155}=m,\;c_{456}=\frac{n}{4} \end{array}\right.$$

For a uniformly predeformed state under axial tension without rigid body rotation, the static displacement gradient is determined from the initial strain given in Equation (2), and the initial stress tensor has only one nonzero component in the axial direction:

(6) $$w_{rr}=w_{\theta \theta }=-\frac{\nu }{E}\sigma_{z},\;w_{zz}=\frac{\sigma_{z}}{E},\;\tau_{zz}^{0}\approx \sigma_{z}$$

All other initial stress components and shear components of the static displacement gradient are zero.

### 2.2. SAFE Formulation for Stress-Dependent Longitudinal Modes

Hayashi et al. applied the semi analytical finite element method to calculate phase and group velocity dispersion curves of guided waves in waveguides with arbitrary cross-sections [22]. The axisymmetric longitudinal modes in a hollow circular tube are denoted by L(0,*m*), where 0 represents the circumferential order and *m* denotes the longitudinal mode branch index [23]. For these modes, all field quantities are independent of the circumferential coordinate *θ*, and the circumferential displacement component is zero.

The semi-analytical finite element (SAFE) analysis is performed in the unloaded reference configuration. The displacement field of an axially propagating longitudinal mode is expressed as

(7) $$u\left(r,z_{R},t\right)=\left[\begin{array}{c} U_{r}\left(r\right)\\ U_{z}\left(r\right) \end{array}\right]e^{i\left(k_{R}z_{R}-\omega t\right)},\;\frac{\partial \left(\cdot \right)}{\partial \theta }=0,\;u_{\theta }=0$$

where $U_{r}\left(r\right)$ and $U_{z}\left(r\right)$ are the radial and axial displacement functions over the cross-section, respectively; $z_{R}$ and $k_{R}$ denote the axial coordinate and axial wavenumber in the reference configuration, respectively; and ω is the angular frequency.

Since the full incremental stiffness is formulated in terms of the dynamic displacement gradient, the displacement gradient of an arbitrary radial element can be written in the unified form

(8) $$H^{\left(e\right)}=\left(ik_{R}G_{1}^{\left(e\right)}+G_{2}^{\left(e\right)}\right)q^{\left(e\right)}e^{i\left(k_{R}z_{R}-\omega t\right)}$$

where $q^{\left(e\right)}$ is the nodal displacement vector containing the radial and axial displacement degrees of freedom, $G_{1}^{\left(e\right)}$ contains the axial derivative terms, and $G_{2}^{\left(e\right)}$ contains the radial derivative terms and the 1/*r* terms arising from the cylindrical coordinate system. For axisymmetric longitudinal modes, the nonzero components of the dynamic displacement gradient are $u_{r,r}$, $u_{r,z}$, $u_{r}/r$, $u_{z,r}$, and $u_{z,z}$.

After the full incremental stiffness in Equation (3) is assembled into the matrix $A^{\sigma }$ according to the arrangement of the dynamic displacement gradient components, the incremental stress within an element can be expressed as

(9) $$s^{\left(e\right)}=A^{\sigma }H^{\left(e\right)}$$

In the absence of body forces and externally applied dynamic surface tractions, the virtual work equation is given by

(10) $$\int_{V}\delta H^{H}s\;dV+\int_{V}\delta u^{H}\rho_{0}\ddot{u}\;dV=0$$

Substituting Equations (8) and (9) into Equation (10) and assembling all radial elements yields the SAFE eigenvalue equation for axisymmetric longitudinal modes [24,25]:

(11) $$\left[k_{R}^{2}K_{1}^{\sigma }+ik_{R}K_{2}^{\sigma }+K_{3}^{\sigma }-\omega^{2}M\right]q=0$$

The corresponding matrices are expressed as

(12) $$\left\{\begin{array}{l} \begin{array}{l} K_{1}^{\sigma }=\int_{r_{i}}^{r_{o}}G_{1}^{H}A^{\sigma }G_{1}r\;dr\\ K_{2}^{\sigma }=\int_{r_{i}}^{r_{o}}\left(-G_{1}^{H}A^{\sigma }G_{2}+G_{2}^{H}A^{\sigma }G_{1}\right)r\;dr \end{array}\\ \begin{array}{l} K_{3}^{\sigma }=\int_{r_{i}}^{r_{o}}G_{2}^{H}A^{\sigma }G_{2}r\;dr\\ M=\int_{r_{i}}^{r_{o}}\rho_{0}N^{H}Nr\;dr \end{array} \end{array}\right.$$

where $r_{i}$ and $r_{o}$ are the inner and outer radii in the reference configuration, respectively, N is the element shape function matrix, and the superscript *H* denotes the conjugate transpose. The common factor 2π arising from the circumferential integration can be cancelled from the eigenvalue equation. The inner and outer surfaces of the tube satisfy the incremental traction free boundary condition:

(13) $$sn=0,\;r=r_{i},\;r_{o}$$

This condition is naturally satisfied in the virtual work formulation.

For a given reference wavenumber $k_{R}$, solving Equation (11) yields the dispersion relations of the longitudinal modes. The phase and group velocities in the reference configuration are defined as

(14) $$c_{p}^{R}=\frac{\omega }{k_{R}},c_{g}^{R}=\frac{d\omega }{dk_{R}}$$

The ordering of eigenvalues at adjacent wavenumber points does not necessarily correspond to the same physical mode. Therefore, the modes are tracked between adjacent wavenumber points based on eigenvector correlation and the continuity of the dispersion branches [26]. The propagation velocity in a prestressed medium depends on the configuration in which it is defined. The velocities obtained from Equation (14) are defined with respect to the unloaded reference configuration, whereas the experimentally measured propagation distance is defined in the current configuration after loading. Under uniform axial tension, the reference and current axial coordinates satisfy

(15) $$z_{C}=\lambda_{z}z_{R},\;\lambda_{z}\approx 1+\frac{\sigma_{z}}{E},\;k_{C}=\frac{k_{R}}{\lambda_{z}}$$

Accordingly, the phase and group velocities in the current configuration are given by

(16) $$c_{p}^{C}=\lambda_{z}c_{p}^{R},{\;c}_{g}^{C}=\lambda_{z}c_{g}^{R}$$

The phase and group velocities in the current configuration obtained above are used in the subsequent stress sensitivity analysis and target mode selection.

### 2.3. Stress Sensitivity of Longitudinal Modes and Evaluation of the L(0,2) Operating Frequency

**Figure 1:**
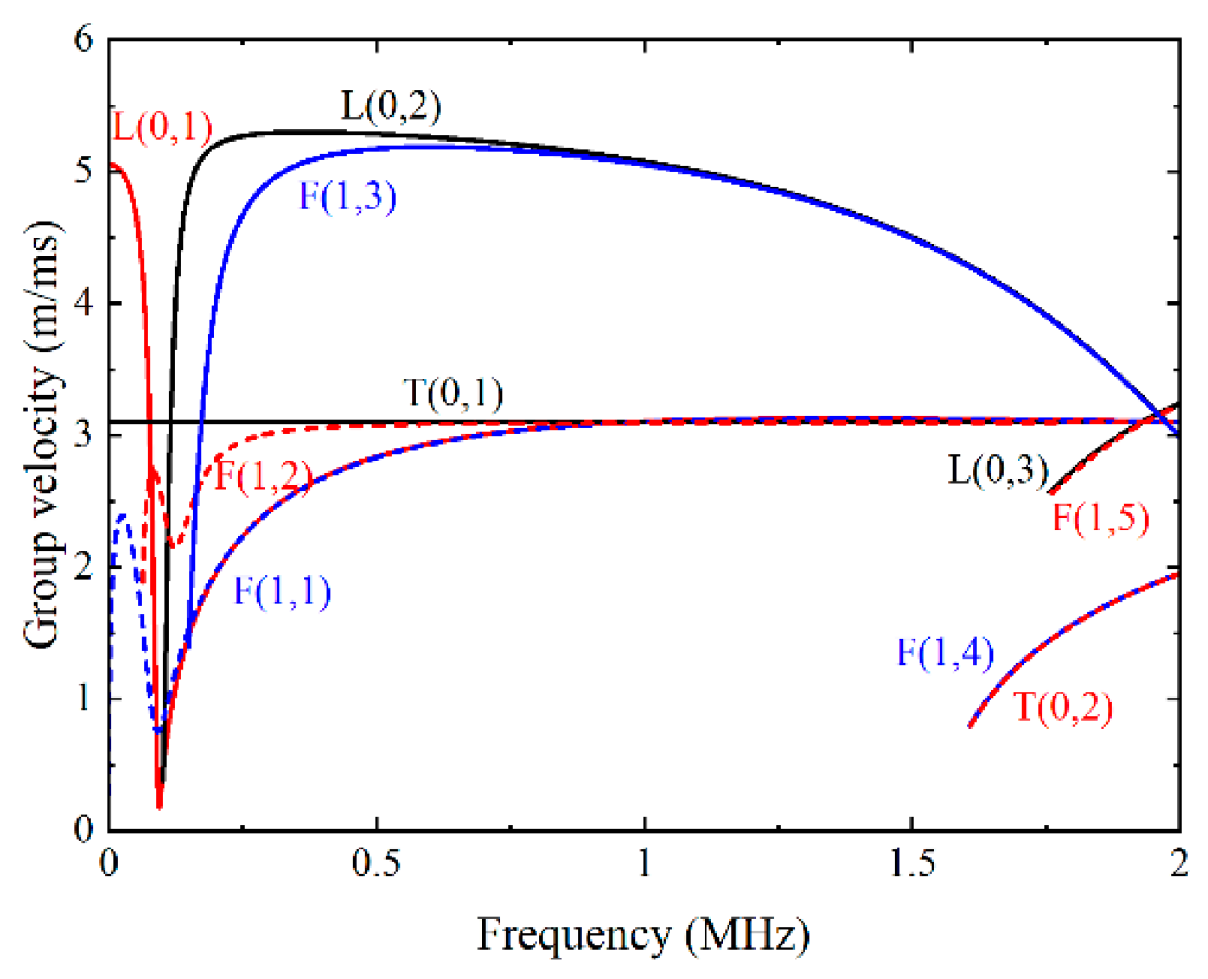
Dispersion curves of guided wave modes in the aluminum alloy tube with an outer diameter of 18 mm and a wall thickness of 1 mm under the stress-free condition.

The dispersion curves of all guided wave modes in the hollow aluminum alloy tube under the stress-free condition are shown in Figure 1. The L(0,2) mode exhibits a relatively high group velocity within the candidate frequency band and can therefore serve as an early arriving component at the receiving end. This characteristic facilitates temporal separation of the L(0,2)-dominated wave packet from slower modes such as L(0,1) and T(0,1). However, Figure 1 also shows that the group velocity of the F(1,3) mode approaches that of L(0,2) around 500 kHz. Therefore, arrival time alone is insufficient to distinguish L(0,2) from F(1,3) at this frequency. At approximately 250 kHz, a larger group velocity separation exists between these two modes. This difference is considered explicitly in the transient response analysis in Section 3.2.

**Figure 2:**
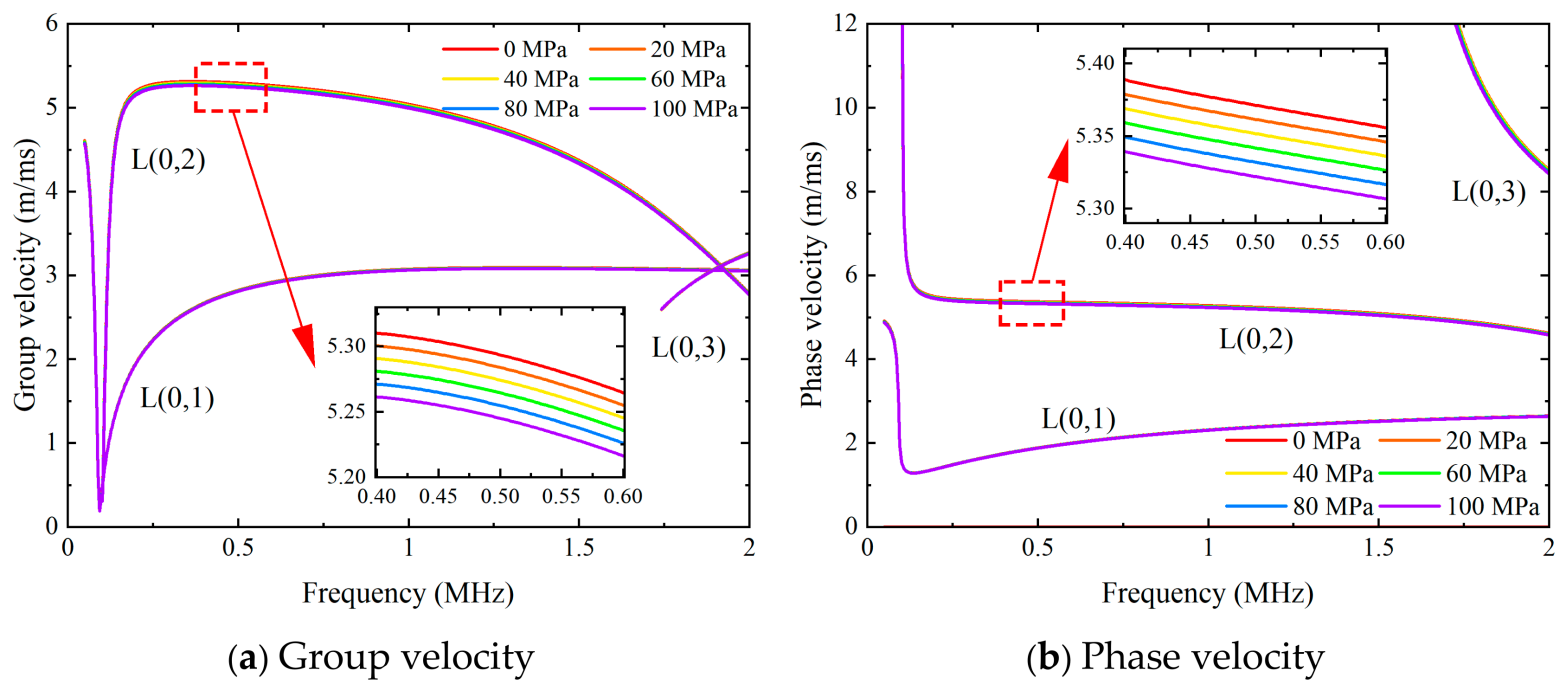
Group and phase velocity dispersion curves of longitudinal modes under different axial tensile stresses.

To quantify the effect of axial prestress, the uniform axial tensile stress $\sigma_{z}$ was varied from 0 to 100 MPa in increments of 20 MPa. For each prescribed stress level, the corresponding initial strain and prestress-dependent effective stiffness were updated according to the formulation in Section 2.1, and the SAFE eigenvalue problem was solved accordingly. The resulting group and phase velocity dispersion curves of the longitudinal modes for the 18 mm outer diameter and 1 mm wall thickness tube are shown in Figure 2 to evaluate the stress-induced velocity variations. The target mode for TOF based stress measurement should remain sufficiently far from mode cutoff regions and exhibit low group velocity dispersion within the effective frequency band of the probe. These characteristics help reduce wave packet broadening and waveform distortion during the propagation of finite bandwidth signals [27].

The dispersion results show that the group velocity curve of the L(0,2) mode is relatively flat within 0.40–0.60 MHz, with a group velocity of approximately 5.2–5.3 m/ms. Its group velocity is substantially higher than that of the L(0,1) mode within the same frequency range. Therefore, the L(0,2)-dominated wave packet can arrive at the receiving end earlier and maintain a certain degree of temporal separation from the subsequent slower longitudinal modes.

As the axial tensile stress increases, both the phase and group velocities of the L(0,2) mode gradually decrease. The stress-induced velocity variations caused by the acoustoelastic effect are much smaller than the absolute guided wave velocities. Consequently, the dispersion curves corresponding to different stress states remain close to each other over the full frequency range shown. The enlarged views are therefore used to illustrate the stress-induced velocity shifts within 0.40–0.60 MHz.

At each frequency, the relationships between propagation velocity and axial stress are fitted linearly, with the stress-free phase and group velocities fixed as the corresponding intercepts [28]:

(17) $$c_{p}\left(f,\sigma_{z}\right)=c_{p}^{0}\left(f\right)+a_{p}\left(f\right)\sigma_{z},\;c_{g}\left(f,\sigma_{z}\right)=c_{g}^{0}\left(f\right)+a_{g}\left(f\right)\sigma_{z}$$

where $c_{p}^{0}\left(f\right)$ and $c_{g}^{0}\left(f\right)$ are the phase and group velocities under the stress-free condition, respectively, and $a_{p}\left(f\right)$ and $a_{g}\left(f\right)$ are the corresponding fitted slopes with respect to axial stress.

The normalized stress sensitivity coefficients of the phase and group velocities are defined as

(18) $$K_{p}\left(f\right)=\frac{a_{p}\left(f\right)}{c_{p}^{0}\left(f\right)},\;K_{g}\left(f\right)=\frac{a_{g}\left(f\right)}{c_{g}^{0}\left(f\right)}$$

where $K_{p}$ and $K_{g}$ both have units of $MPa^{-1}$. $K_{p}$ characterizes the stress response of the phase velocity and the associated oblique incidence phase-matching condition, whereas $K_{g}$ characterizes the stress response of the group velocity and the corresponding wave packet TOF.

According to Equations (15) and (16), under the first-order acoustoelastic approximation, the stress sensitivity coefficients in the current configuration are related to the SAFE results in the reference configuration by

(19) $$K_{p}\approx K_{p}^{R}+\frac{1}{E},\;K_{g}\approx K_{g}^{R}+\frac{1}{E}$$

**Figure 3:**
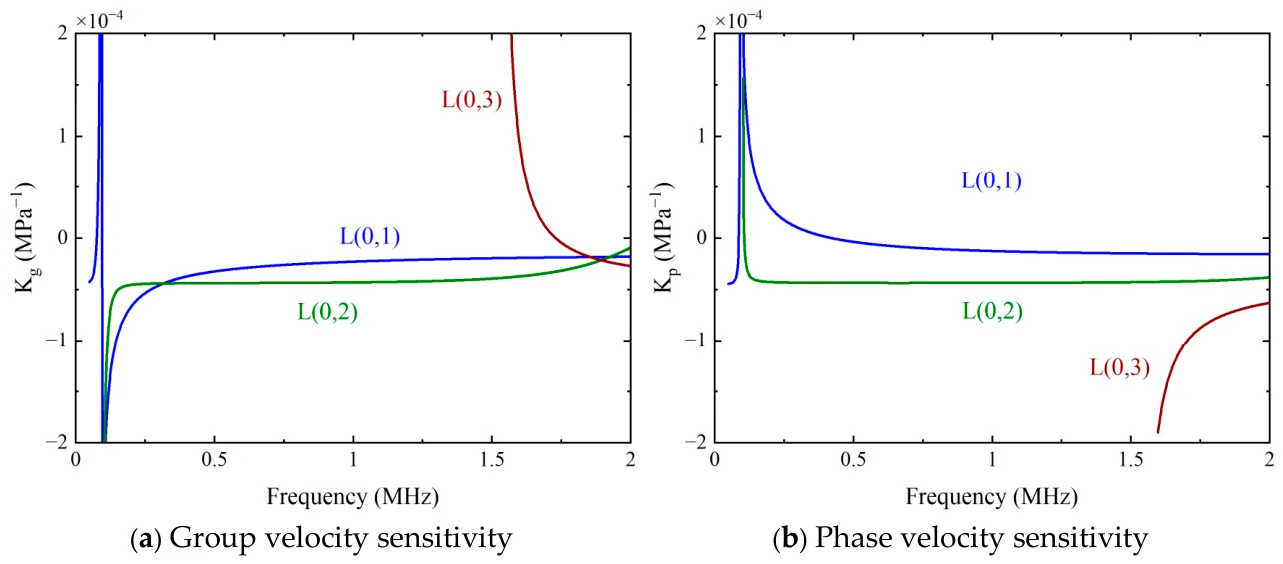
Normalized stress sensitivity coefficients of longitudinal modes.

The phase and group velocity stress sensitivity coefficients of the longitudinal modes are shown in Figure 3. Near 500 kHz, both $K_{p}$ and $K_{g}$ of the L(0,2) mode are negative and vary only slightly with frequency. The negative values indicate that increasing axial tensile stress decreases both the phase and group velocities of this mode. The calculated normalized group velocity stress sensitivity coefficient of the L(0,2) mode in the current configuration at 500 kHz is approximately $-4.37\times 10^{-5}\;MPa^{-1}$.

Relatively large local stress sensitivity coefficients may occur near mode cutoff regions or turning regions of the dispersion curves. However, these regions are generally accompanied by strong dispersion and rapidly varying group velocity, which are unfavorable for stable propagation of a finite bandwidth wave packet and TOF extraction. Therefore, the suitability of an operating frequency cannot be evaluated solely from the maximum stress sensitivity coefficient or the group velocity separation between individual modes [29].

At approximately 250 kHz, L(0,2) exhibits a larger group velocity separation from the nearby F(1,3) mode than at 500 kHz. In contrast, L(0,2) exhibits relatively low dispersion and stable stress sensitivity around 500 kHz. Because the L(0,2) and F(1,3) group velocities are close, near 500 kHz, the target response cannot be identified solely from its arrival time. The finite duration responses at 250 and 500 kHz are therefore further compared in Section 3.2, while the circumferential characteristics of the target wave packet are examined in Section 3.3.

### 2.4. Relationship Between TOF Shift and Axial Stress

In the experiments, the TOF of the target wave packet between the transmitting and receiving probes is measured [30]. The target wave packet has a center frequency of 500 kHz. When the wave packet has a narrow bandwidth, the group velocity varies only slightly within the operating frequency band, and the wave packet shape remains approximately unchanged before and after loading, its propagation velocity can be approximated by the group velocity at the center frequency. Thus,

(20) $$f_{c}=500\;kHz,\;c_{g,0}=c_{g}^{0}\left(f_{c}\right),\;K_{g}=K_{g}\left(f_{c}\right)$$

where $c_{g,0}$ is the stress-free group velocity at the center frequency, and $K_{g}$ is the normalized group velocity stress sensitivity coefficient at the center frequency.

According to Equations (17) and (18), the group velocity under an axial stress $\sigma_{z}$ can be expressed as

(21) $$c_{g}\left(\sigma_{z}\right)=c_{g,0}\left(1+K_{g}\sigma_{z}\right)$$

In the experimental setup, the positions of the transmitting and receiving probes are constrained by an external fixture, and the corresponding equivalent acoustic interaction positions on the tube wall remain unchanged during each loading test. Therefore, the effective propagation distance in the theoretical model satisfies

(22) $$L\left(\sigma_{z}\right)=L_{0}$$

Under an axial stress $\sigma_{z}$, the propagation time of the target wave packet is given by

(23) $$t\left(\sigma_{z}\right)=\frac{L_{0}}{c_{g,0}\left(1+K_{g}\sigma_{z}\right)}$$

The propagation time at zero stress is

(24) $$t_{0}=\frac{L_{0}}{c_{g,0}}$$

When $\left|K_{g}\sigma_{z}\right|\ll 1$, a first-order expansion of Equation (23) with respect to axial stress gives the theoretical TOF shift as

(25) $$\Delta t_{th}=t\left(\sigma_{z}\right)-t_{0}\approx -t_{0}K_{g}\sigma_{z}=K_{t}^{th}\sigma_{z},\;K_{t}^{th}=-t_{0}K_{g}$$

Since $K_{g}<0$ for the L(0,2) mode near 500 kHz, $K_{t}^{th}>0$. This indicates that increasing axial tensile stress decreases the group velocity of the target wave packet and increases its TOF.

The corresponding theoretical relationship for axial stress estimation is

(26) $$\sigma_{z}=\frac{\Delta t_{th}}{K_{t}^{th}}$$

This relationship links the group velocity stress sensitivity coefficient obtained from the dispersion analysis to the TOF shift measured from the experimental signals. The received signals also contain fixed propagation delays introduced by the piezoelectric elements, polymethyl methacrylate (PMMA) wedges, coupling layers, and acquisition system. These stress-independent delays are cancelled in the relative TOF calculation between the loaded signal and the zero-load reference signal.

## 3. Conformal Oblique Incidence Probe Design and Guided Wave Response Analysis

### 3.1. Design of the Conformal Oblique Incidence-Guided Wave Probe

**Figure 4:**
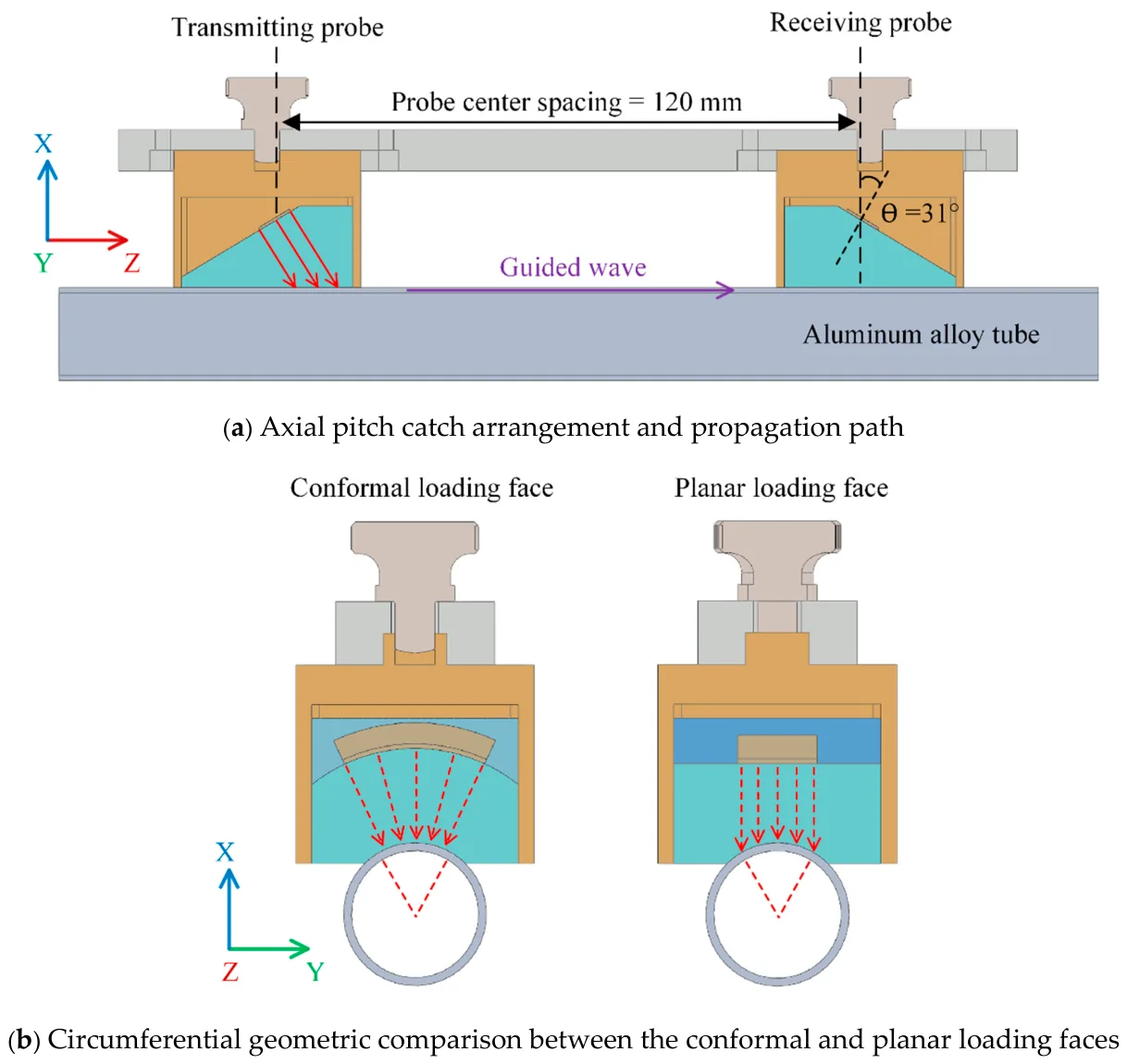
Conformal probe design and loading configuration. The red arrows indicate incident wave directions, while the purple arrow indicates axial guided wave propagation.

A pitch catch configuration is adopted for guided wave inspection. As shown in Figure 4a, the transmitting and receiving probes are mounted on the outer surface of the aluminum alloy tube and aligned along the tube axis, with a probe center spacing of 120 mm. The oblique incidence direction is oriented along the tube axis to generate the target guided wave response along the tube axis. This arrangement provides a fixed probe center spacing for subsequent guided wave propagation and TOF measurements.

As shown in Figure 4b, the ultrasonic waves generated by the piezoelectric element are coupled into the tube wall through a wedge. The probe has a center frequency of 500 kHz, and the bottom surface of the wedge is machined into a concave profile that matches the outer surface of the 18 mm diameter tube. Although a conventional planar piezoelectric oblique incidence probe can conform to the curved tube surface through a curved wedge, the effective loading face associated with the planar piezoelectric element remains approximately planar. For a small diameter tube, the geometric mismatch between the planar loading face and the local curvature of the tube wall causes variations in the incidence direction and equivalent loading distribution over the limited circumferential region. To mitigate this mismatch, the piezoelectric element and its corresponding loading face are designed with a conformal curvature along the circumferential direction, so that the loading face matches the local curvature of the tube wall over the limited circumferential region. This design is intended to improve the consistency of the circumferential loading distribution and provide more favorable excitation conditions for enhancing an L(0,2)-dominated response with more pronounced axisymmetric characteristics.

The oblique incidence angle is determined from the phase-matching relationship between the longitudinal wave velocity in the wedge and the phase velocity of the target guided wave mode. For the L(0,2) mode at a center frequency of 500 kHz, the oblique incidence relationship is expressed as

(27) $$\sin\theta =\frac{c_{w}}{c_{p,0}}$$

where θ is the angle between the incident wave beam in the wedge and the normal to the tube wall, $c_{w}$ is the longitudinal wave velocity in the wedge material, and $c_{p,0}$ is the stress-free phase velocity of the L(0,2) mode at 500 kHz. Based on the phase velocity obtained in Section 2 and the longitudinal wave velocity of the wedge material, the calculated incidence angle is approximately 31°. Therefore, the wedge incidence angles of both the transmitting and receiving probes are set to 31°.

Compared with circumferentially distributed piezoelectric elements, ring arrays, or multiple probe arrangements, the proposed conformal probe achieves local oblique incidence excitation over only a limited circumferential region and does not require transducer elements to be arranged around the entire tube circumference. It is therefore more suitable for installation and inspection in the restricted spaces encountered in small diameter aircraft tubing. While maintaining a compact installation configuration, local curvature matching can improve the circumferential excitation conditions. The design aims to enhance the L(0,2)-dominated target wave packet and increase the time domain prominence of the target wave packet relative to subsequent responses, thereby facilitating the extraction of small TOF shifts and subsequent axial stress measurement. The effects of the conformal structure on the wavefield propagation characteristics and the identifiability of the target wave packet are further evaluated through three-dimensional finite element analysis and experiments.

### 3.2. Finite Element Model and Transient Response Analysis

The SAFE analysis in Section 2 provides the dispersion and stress sensitivity characteristics of guided wave eigenmodes in an ideal uniform waveguide, but it does not describe the finite duration wave packets generated by local oblique incidence excitation. A three-dimensional transient finite element model was therefore established to examine the time domain characteristics of the candidate operating frequencies. The electromechanical coupling process of the piezoelectric element was not modeled explicitly. Instead, the excitation was represented by an equivalent time varying uniform pressure applied normal to the loading face. A five-cycle Hanning windowed sinusoidal signal was used as the excitation. The aluminum alloy tube was discretized using C3D8R three-dimensional solid elements, with eight elements distributed through the wall thickness. The received displacement signal was extracted from the outer surface node corresponding to the center of the receiving probe. The dispersion analysis in Section 2.3 shows that L(0,2) has a substantially larger group velocity separation from F(1,3) at approximately 250 kHz than at 500 kHz. However, over the present 120 mm propagation distance, the corresponding theoretical arrival time difference at 250 kHz is approximately 2.8 μs. For the five-cycle excitation used in the finite element model, the excitation duration is 20 μs at 250 kHz and 10 μs at 500 kHz. Therefore, although the larger group velocity separation at 250 kHz is favorable in terms of modal arrival time, the resulting time difference remains small relative to the finite excitation duration over the present short propagation distance.

**Figure 5:**
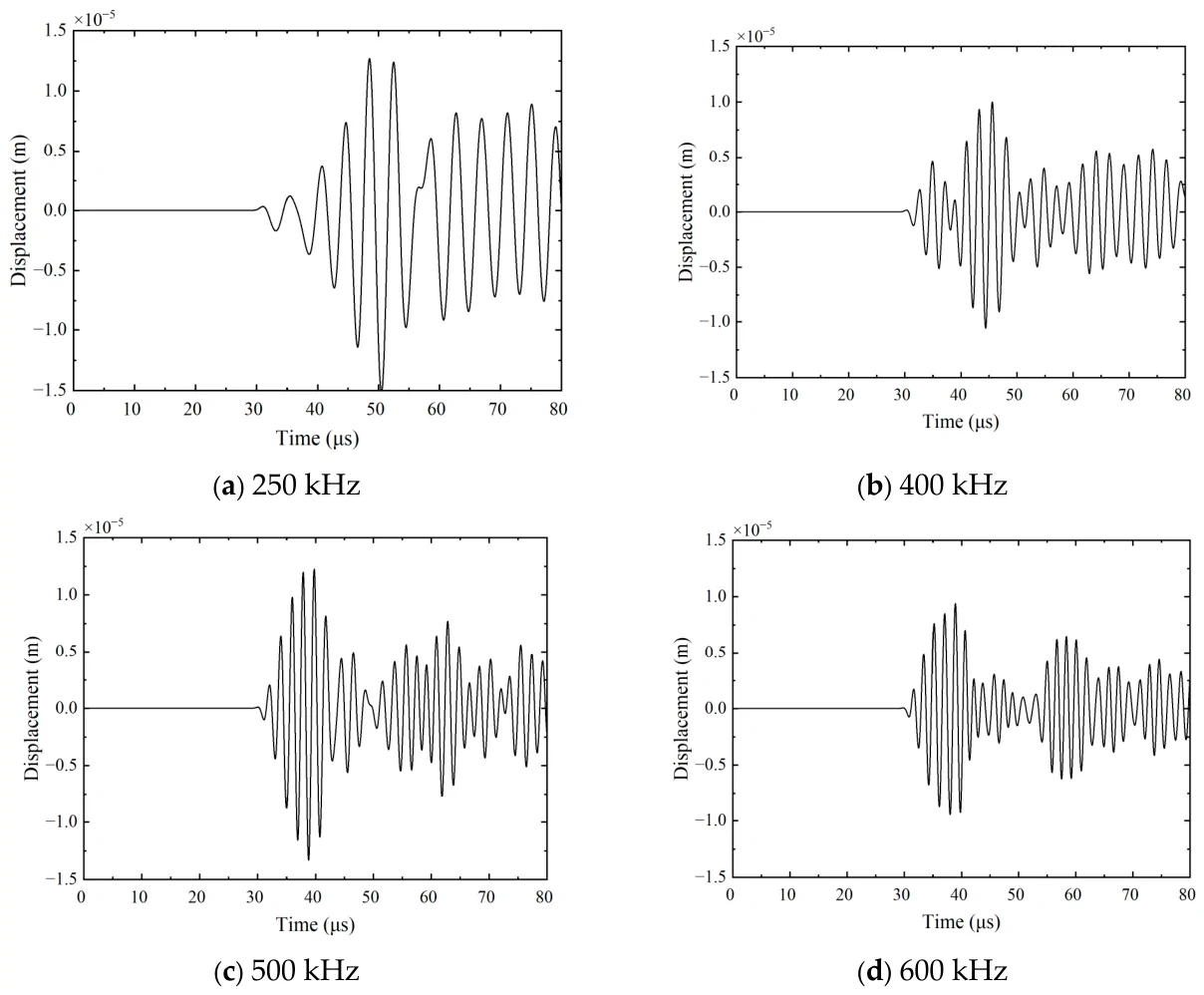
Received signals under 250, 400, 500, and 600 kHz excitation.

As shown in Figure 5, the early arriving response at 250 kHz extends over a broader time interval, whereas the corresponding response at 500 kHz is more temporally concentrated. This result is consistent with the above time scale comparison and shows that the larger group velocity separation at 250 kHz does not necessarily provide better temporal separation of the finite duration responses under the present propagation conditions. The responses at 400, 500, and 600 kHz were further compared within the 0.40–0.60 MHz region identified in Section 2, where L(0,2) exhibits relatively low dispersion and stable stress sensitivity. At 400 kHz, the early arriving response is distributed over a broader time interval, indicating more evident wave packet broadening. At 600 kHz, a clearer low amplitude interval appears between the early arriving and subsequent responses, whereas the 500 kHz response remains comparatively concentrated in time. These results provide a local assessment of the transient response characteristics around the 500 kHz candidate frequency.

Considering the relatively low dispersion and stable stress sensitivity of L(0,2), around 500 kHz, together with the transient response characteristics, 500 kHz was selected as the operating frequency for the subsequent probe configuration analysis and experimental investigation. Because the group velocities of L(0,2) and F(1,3) remain close near this frequency, the circumferential characteristics of the selected target response are further examined in Section 3.3.

### 3.3. Circumferential Characteristics and Identifiability of the L(0,2)-Dominated Wave Packet

After adopting 500 kHz as the operating frequency for the present sensing configuration, the circumferential characteristics of the received guided wave responses generated by the conventional planar probe and the conformal probe were further examined. Since the excitation is introduced through a local oblique incidence configuration over a limited circumferential region, the generated wave field is expected to contain a superposition of multiple guided wave components rather than an ideal single eigenmode. Therefore, the target response is identified as an L(0,2)-dominated wave packet based on the combined evidence of phase-matching conditions, propagation characteristics, and circumferential waveform consistency. Because the two loading faces have different areas, the excitation pressures were scaled according to their respective loading face areas to provide the same surface integrated pressure magnitude. The loading face areas were 53.2 mm^2^ for the planar probe and 147.29 mm^2^ for the conformal probe, resulting in a pressure amplitude ratio of approximately 2.77:1. The probe center spacing, excitation position, receiving position, excitation waveform, and other computational parameters were kept unchanged. The planar probe employed a planar equivalent loading face, whereas the conformal probe employed a conformal equivalent loading face matching the local curvature of the tube wall.

**Figure 6:**
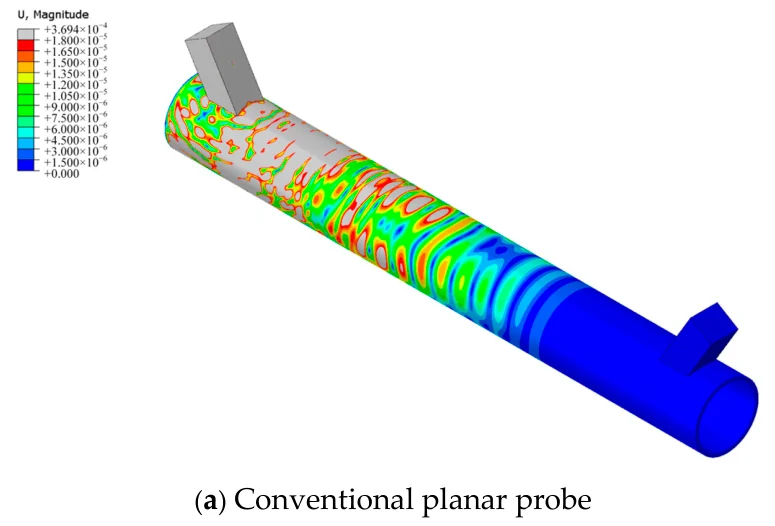

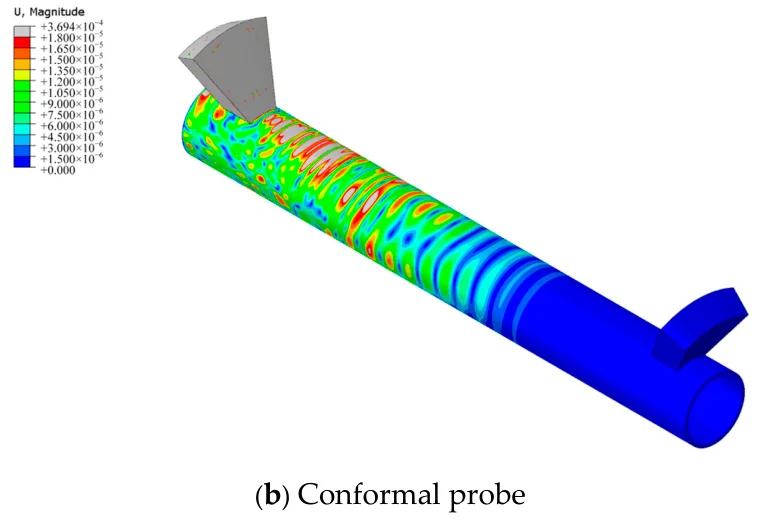
Simulated wavefields of the planar and conformal probes at 500 kHz.

Figure 6 shows the transient displacement wavefields on the tube wall at 24 μs under 500 kHz excitation. Both loading configurations generate a dominant response propagating along the tube axis. The simulated wavefield represents the spatial superposition of guided wave components generated by the local oblique excitation. Therefore, the comparison focuses on the propagation characteristics and circumferential consistency of the target response rather than strict modal energy decomposition. After scaling the excitation pressures according to the respective loading face areas, continuous propagating wavefronts are observed for both configurations. However, differences remain in their circumferential spatial distributions. The planar loading configuration produces a more oblique and circumferentially nonuniform wavefield, whereas the conformal loading configuration exhibits a more regular circumferential distribution. Since the surface-integrated pressure magnitude was kept the same for the two configurations, these differences mainly reflect the spatial loading distributions associated with the two probe geometries. Therefore, the simulation comparison focuses on the circumferential distribution and waveform characteristics rather than absolute displacement amplitude.

Because the group velocities of L(0,2) and F(1,3) are close, near 500 kHz, arrival time alone is insufficient to distinguish these two components. Therefore, the circumferential waveform characteristics of the selected target response were further examined using the axial displacement U1 extracted at 0°, 90°, 180°, and 270° on the same tube cross-section. Since the tube axis coincides with the global X direction in the finite element model, U1 represents the axial displacement component. Considering that the local oblique excitation generates a nonuniform circumferential wavefield, the analysis focused on the waveform consistency between different circumferential positions rather than an ideal modal decomposition. The phase relationship and waveform similarity between opposite circumferential positions were examined to determine whether the selected response exhibited the characteristic phase reversal expected from a dominant first-order flexural component.

For the conformal probe, the target responses at 0° and 180° exhibited highly consistent waveform characteristics, with a normalized correlation coefficient of 0.976. The responses at 90° and 270° were also highly consistent, with a normalized correlation coefficient of 1.000. For the planar probe, the corresponding correlation coefficient between the 0° and 180° responses was 0.857. These results indicate that the selected target response does not exhibit the opposite phase relationship typically associated with a dominant first-order flexural component. Instead, the response maintains a relatively consistent axial displacement characteristic around the measured circumferential positions. The improved circumferential waveform consistency obtained with the conformal configuration is also consistent with the more regular circumferential wavefield distribution observed in Figure 6. Together with the 31° phase-matching condition obtained from the SAFE dispersion analysis, the corresponding group velocity behavior and circumferential waveform consistency support identifying the selected response as an L(0,2)-dominated response among the possible guided wave components considered in this study.

## 4. Experimental Validation and Discussion

### 4.1. Axial Loading and Guided Wave Measurement System

**Figure 7:**
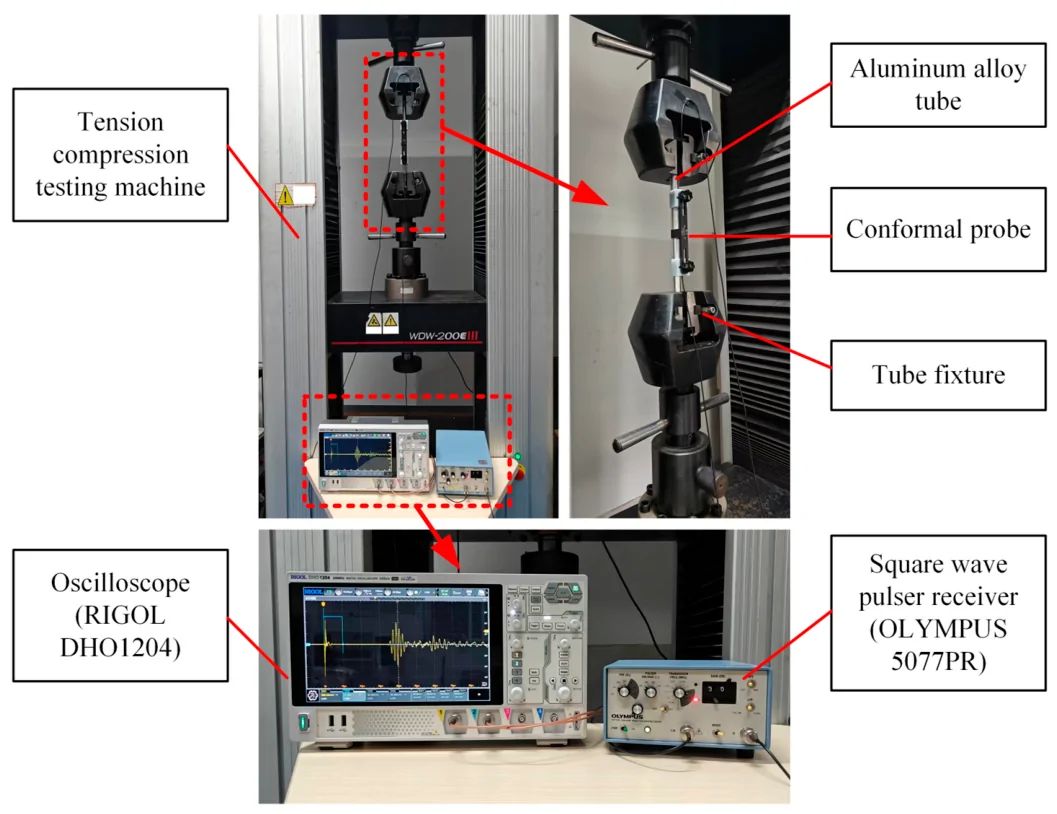
Experimental system for axial loading and guided wave measurement.

Axial loading experiments were conducted to evaluate the axial stress measurement performance of the conformal oblique incidence-guided wave probe in small diameter thin-walled aluminum alloy tubes. Additional comparative experiments using a conventional planar probe were conducted under the same loading and signal acquisition conditions. The experimental system is shown in Figure 7. To maintain the cross-sectional integrity of the thin-walled tube during gripping, cylindrical alloy steel mandrels were inserted into both tube ends before clamping. The mandrels provided internal radial support and reduced local collapse and excessive ovalization of the tube wall caused by the wedge grips. Before the formal measurements, the tube axis was carefully aligned with the centers of the upper and lower grips and with the loading axis of the testing machine. A preliminary loading and unloading cycle was then performed by increasing the axial stress to 100 MPa and subsequently unloading the specimen until the measured tensile force returned to zero. This preconditioning procedure reduced initial seating related mechanical variations of the specimen and gripping system and reduced the influence of initial mechanical slack during the subsequent loading tests. The zero-load reference waveform was acquired after unloading while the specimen remained fully clamped in the same fixture configuration.

The transmitting and receiving probes were symmetrically positioned within the straight free section of the specimen, with the nearest edge of each probe tube contact footprint located at least 40 mm from the corresponding gripping boundary. This arrangement reduced the influence of local gripping-induced disturbances on the guided wave measurement region. The tensile force was measured using the load measurement system of the WDW-200E III universal testing machine (Jinan Shidai Shijin Testing Machine Co., Ltd., Jinan, China), which is rated as Class 1. The axial stress calculated from the measured tensile force and the initial tube cross-sectional area according to Equation (1) was used as the reference stress for calibration and validation. Guided wave signals were acquired only after each prescribed load level had reached a stable state.

The guided wave measurement system consisted of an OLYMPUS 5077PR square wave pulser receiver (Olympus NDT, Inc., Waltham, MA, USA), a RIGOL DHO1204 digital oscilloscope (RIGOL Technologies Co., Ltd., Suzhou, China), and a pair of conformal oblique incidence-guided wave probes. The transmitting and receiving probes were mounted on the outer surface of the aluminum alloy tube in a pitch catch arrangement along the tube axis. The probe center spacing was 120 mm. The transmitting and receiving probes were constrained by an external positioning fixture, so their center spacing remained fixed at 120 mm throughout each loading test and did not increase with the axial elongation of the specimen. The probes had a center frequency of 500 kHz and a wedge incidence angle of 31°. For the comparative experiments, a conventional planar probe pair with the same nominal center frequency of 500 kHz and wedge incidence angle of 31° was used with the same pitch catch arrangement and probe center spacing. The bottom surface of each wedge was machined into a concave profile matching the outer surface of the 18 mm diameter tube to improve contact with the curved tube surface. Industrial oil was used as the couplant.

The OLYMPUS 5077PR was used to generate the excitation pulse and drive the transmitting probe while also amplifying the ultrasonic signal from the receiving probe. In the pitch catch configuration, the transmitting probe was connected to the T/R port and the receiving probe to the R port. The pulse voltage was set to 100 V, the transducer frequency matching setting was 0.5 MHz, and the receiver gain was set to 30 dB. The 0.5 MHz frequency setting corresponds to the 500 kHz center frequency of the probes used in this study. The received signals were acquired using a RIGOL DHO1204 digital oscilloscope. The same acquisition parameters were used for all stress states, with a sampling rate of 1 GSa/s, a memory depth of 100 kpts, and waveform averaging over 16 repeated acquisitions to reduce uncorrelated random noise and improve the signal to noise ratio, thereby stabilizing the subsequent TOF estimation. The raw waveform data were exported using the oscilloscope storage function for subsequent identification of the L(0,2)-dominated target wave packet and extraction of the TOF shift.

To reduce the influence of ambient temperature variations on guided wave velocity and TOF measurements, all loading and guided wave measurements were conducted in a temperature-controlled laboratory. During each loading test, the experimental environment and probe installation conditions were kept stable. Signal acquisition was performed only after each prescribed load level had stabilized to minimize the effects of load fluctuations, slight probe movement, and changes in coupling conditions on the TOF measurements. For repeated tests involving probe removal, reinstallation, and recoupling, a new zero-load reference signal was established under the corresponding test condition. This procedure reduced the influence of fixed TOF differences caused by probe reinstallation and changes in coupling conditions on the subsequent axial stress estimation.

### 4.2. Experimental Comparison of the Conventional Planar and Conformal Probes

**Figure 8:**
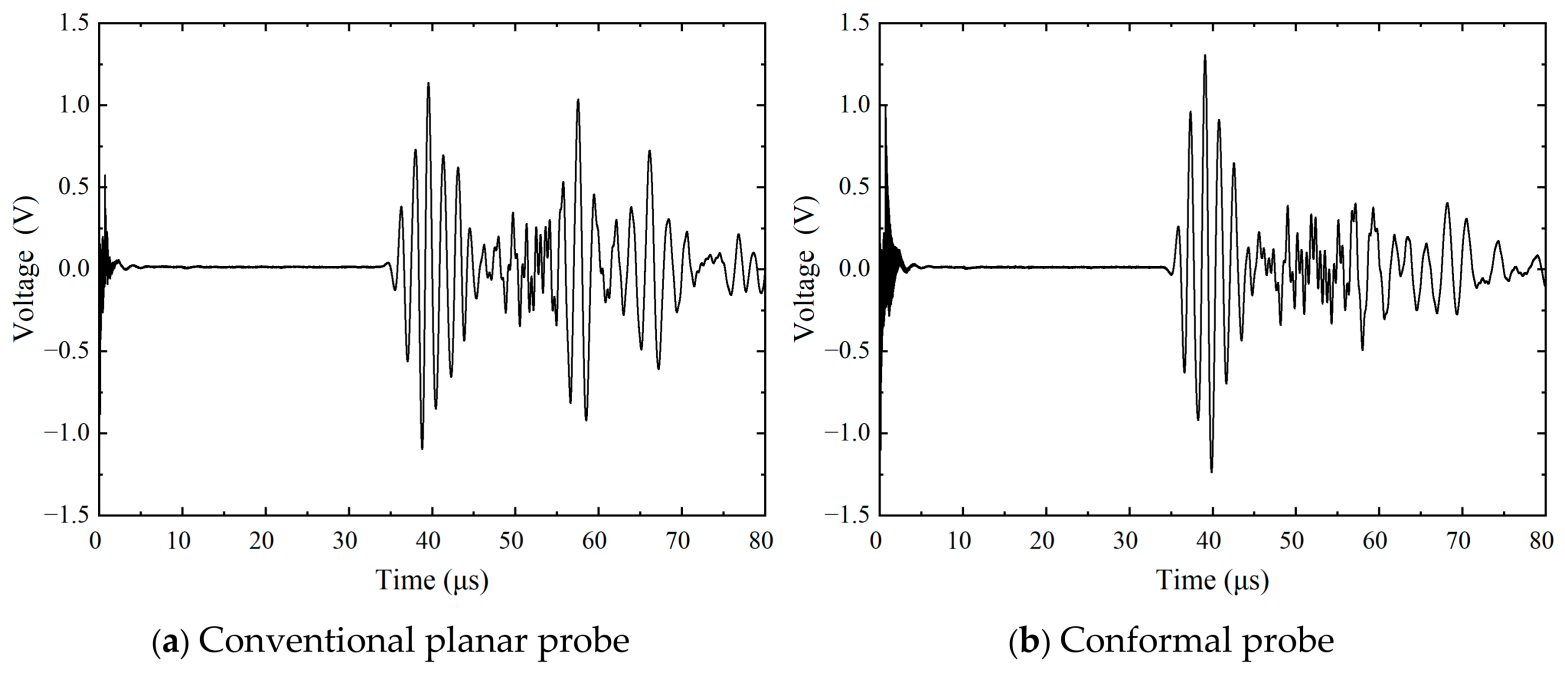
Experimental A scans of the conventional planar and conformal probes at 500 kHz.

To examine whether the above wavefield differences are reflected in the experimental received signals, representative A scan responses obtained using the two probes were further compared at a center frequency of 500 kHz and a wedge incidence angle of 31°, with the same acquisition parameters, as shown in Figure 8. Both probes produce a distinct early arriving target wave packet within approximately 34–44 μs, corresponding to the L(0,2)-dominated response discussed above. However, substantial differences are observed in the subsequent responses. For the conventional planar probe, the peak amplitude of the target wave packet is approximately 1.15 V, whereas a strong subsequent response at approximately 58 μs reaches about 1.05 V, corresponding to approximately 91% of the target wave packet amplitude. In contrast, for the conformal probe, the target wave packet reaches a peak amplitude of approximately 1.3 V, while the subsequent response generally remains below 0.4 V, corresponding to less than 31% of the target wave packet amplitude. Therefore, compared with the absolute signal amplitude, the relative amplitude relationship between the target wave packet and the subsequent response within the same signal more clearly demonstrates that the conformal probe improves the time domain prominence of the target wave packet within the complete received signal.

This experimental observation is consistent with the physical trend indicated by the finite element results. The conventional planar loading configuration produces a wavefield with more pronounced circumferential nonuniformity, while the measured signal contains a strong subsequent response with an amplitude close to that of the target wave packet. In contrast, the conformal loading configuration produces a more regular circumferential wavefield distribution, while the response following the target wave packet is clearly reduced. For subsequent TOF measurements, the significance of this improvement is not simply an increase in received voltage. More importantly, the L(0,2)-dominated target wave packet exhibits clearer temporal boundaries and greater relative prominence. This reduces the sensitivity of target window selection to subsequent propagation components and provides a more stable waveform basis for normalized cross-correlation-based time delay estimation within a narrow time window.

Considering both the finite element wavefields and the experimental received responses, the conformal structure improves the consistency of the circumferential loading distribution on the curved surface of the small diameter tube. The wavefield therefore exhibits a more regular circumferential propagation pattern, while the amplitude ratio of the subsequent response to the target wave packet is clearly reduced. Consequently, the L(0,2)-dominated target wave packet exhibits clearer temporal boundaries and higher identifiability, making it more suitable for the subsequent measurement of small TOF shifts. The influence of these waveform characteristics on TOF based axial stress measurement is further evaluated through the quantitative calibration and validation results in Section 4.4.

### 4.3. TOF Shift Extraction from Experimental Signals

To extract the small TOF variations induced by axial stress, the received signals acquired under different loading states were processed using a unified procedure. Based on the preceding dispersion analysis, finite element results, and the location of the target wave packet in the experimental signals, the time interval of 34–44 μs was selected as the analysis window for the L(0,2)-dominated wave packet. Subsequently arriving wave packets were excluded from the TOF calculation. To eliminate the influence of direct current (DC) offset on the correlation analysis, mean removal was applied to the signals within the analysis window, without any additional smoothing or digital filtering.

The target wave packet under the zero-load condition was used as the reference signal and is denoted by $x_{0}\left[n\right]$. The target wave packet under an axial stress $\sigma_{z}$ is denoted by $x_{\sigma }\left[n\right]$. The TOF shift was extracted using normalized cross-correlation. The normalized cross-correlation function between the zero-load reference wave packet and the wave packet under loading is expressed as

(28) $$R_{0\sigma }\left(m\right)=\frac{\sum_{n}\left[x_{0}\left[n\right]-{\bar{x}}_{0}\right]\left[x_{\sigma }\left[n+m\right]-{\bar{x}}_{\sigma }\right]}{\sqrt{\sum_{n}{\left[x_{0}\left[n\right]-{\bar{x}}_{0}\right]}^{2}\sum_{n}{\left[x_{\sigma }\left[n\right]-{\bar{x}}_{\sigma }\right]}^{2}}}$$

where *m* is the integer lag in samples, and ${\bar{x}}_{0}$ and ${\bar{x}}_{\sigma }$ are the mean values of the reference wave packet and the wave packet under loading within the analysis window, respectively. Although mean removal was applied before the correlation calculation, the general form of the normalized cross-correlation function is retained in Equation (28). The lag corresponding to the maximum of the normalized cross-correlation function was used to determine the TOF shift of the wave packet under loading relative to the zero-load reference wave packet.

To achieve subsample delay estimation, three point parabolic interpolation was applied to the discrete correlation maximum and its two adjacent samples. Assuming that the discrete correlation peak occurs at the *k*th sample of the correlation sequence, with an integer lag of $m_{k}$, and that the corresponding correlation coefficients at the three neighboring samples are $R_{k-1}$, $R_{k}$, and $R_{k+1}$, the subsample correction of the correlation peak is given by

(29) $$\delta =\frac{1}{2}\frac{R_{k-1}-R_{k+1}}{R_{k-1}-2R_{k}+R_{k+1}}$$

The corresponding TOF shift is then calculated as

(30) $$\Delta t=\frac{m_{k}+\delta }{f_{s}}$$

where $f_{s}$ is the sampling rate. According to the sign convention adopted in this study, Δt>0 indicates that the target wave packet under loading is delayed relative to the zero-load reference wave packet. Therefore, the measured TOF shift represents the stress-induced change relative to the reference state. Three-point parabolic interpolation estimates the local peak position around the discrete correlation maximum, enabling subsample delay estimation and reducing the limitation imposed by the sampling interval on TOF shift determination.

Compared with directly tracking a single oscillation peak, normalized cross-correlation determines the relative time shift from the similarity of the entire target wave packet. This reduces the influence of local amplitude variations and fluctuations of individual peaks on TOF determination. The extracted Δt values were subsequently used to calibrate the experimental TOF stress sensitivity coefficient and estimate the axial stress.

### 4.4. Experimental Calibration and Validation of Axial Stress Measurement

**Figure 9:**
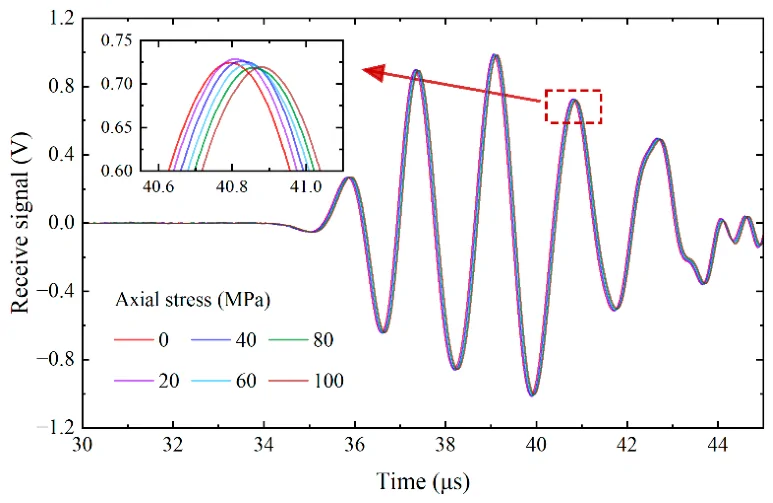
Guided wave signals and TOF shifts under different axial stresses.

The received guided wave signals under different axial tensile stresses are shown in Figure 9. The waveforms obtained at 0, 20, 40, 60, 80, and 100 MPa are presented together with an enlarged view of the target wave packet. As the axial tensile stress increases, the overall waveform shape remains generally consistent, while the target wave packet gradually shifts toward a later arrival time. The enlarged view further shows a progressive and clearly distinguishable TOF shift of the target wave packet. This trend is consistent with the theoretical result in Section 2 that the normalized group velocity stress sensitivity coefficient of the L(0,2) mode in the current configuration is negative. An increase in axial tensile stress decreases the group velocity and increases the TOF. Therefore, the TOF shift of the target wave packet is used as the characteristic parameter for subsequent axial stress estimation.

To establish a quantitative experimental relationship between TOF shift and axial stress, five repeated calibration tests were performed on the same calibration specimen. Each test used its own zero-load state as the reference. The TOF shifts under different stress states were extracted using the normalized cross-correlation method described in Section 4.3. The calibration stress range was 0–100 MPa at 20 MPa intervals.

**Figure 10:**
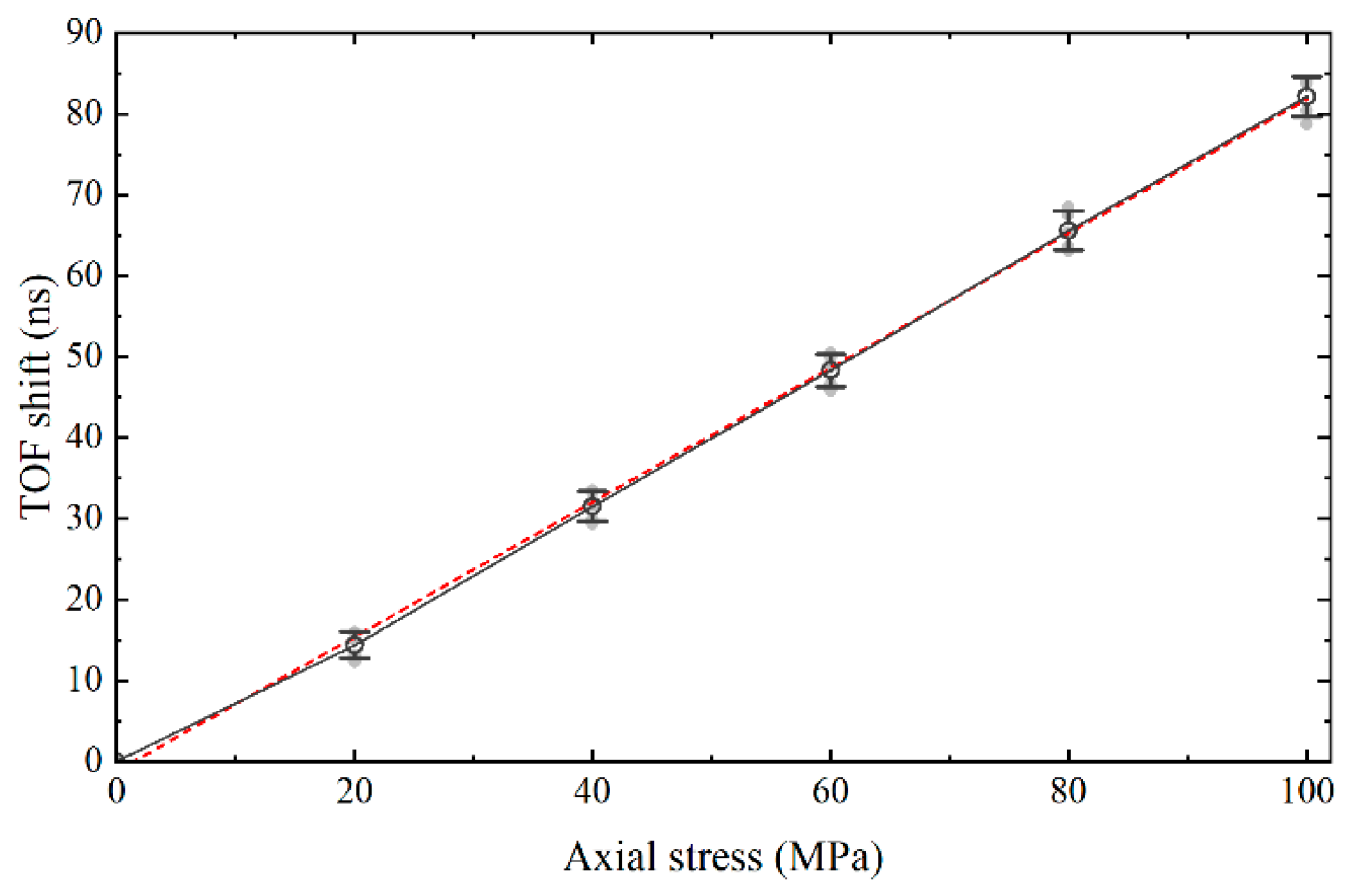
Experimental calibration relationship between TOF shift and axial stress. The solid line connects the mean values, while the dashed line represents the linear fit.

The TOF stress sensitivity coefficients obtained from the five repeated calibration tests were 0.8458, 0.8143, 0.8035, 0.8518, and 0.8352 ns/MPa. The mean value was 0.8301 ns/MPa, with a standard deviation of 0.0206 ns/MPa and a coefficient of variation of 2.48%. Figure 10 shows the relationship between TOF shift and axial stress obtained from the five repeated calibration tests. The gray scatter points represent the individual calibration results, while the hollow markers and error bars represent the mean and standard deviation of the TOF shifts at each stress level, respectively. The red dashed line represents the linear regression result. The TOF shift increases approximately linearly with axial stress, with a coefficient of determination of $R^{2}=0.9994$ and a slope of 0.8301 ns/MPa. This slope is defined as the experimental TOF stress sensitivity coefficient of the L(0,2)-dominated wave packet under the present experimental conditions.

The quantitative validation results were obtained from two additional aluminum alloy tubes from the same batch that were not involved in the calibration. Three validation conditions were considered. Test condition 1 corresponded to the initial test of Tube 1. Test condition 2 was a repeated test of Tube 1 after probe removal, reinstallation, and recoupling. Test condition 3 was an independent test of Tube 2. The validation stress levels were 30, 50, 70, and 90 MPa.

Three repeated signal acquisitions were performed at each stress level. For each test condition, the three waveforms acquired at the initial zero-load state were averaged pointwise to establish the reference signal. The present method is reference based and determines the axial stress change relative to a reference state. In the present experiments, the zero applied load state was used as the reference, so that the measured TOF shift corresponded to the applied axial stress relative to this baseline. When the axial stress of the reference state is known, the axial stress at a subsequent state can be determined by combining the known reference stress with the stress change obtained from the calibrated TOF stress relationship. If no reference state with a known stress is available, the present formulation cannot independently determine the pre-existing absolute axial stress. For practical applications to aircraft tubing, a baseline waveform may be acquired before installation while the tube is in an unloaded and unconstrained condition, allowing subsequent assembly-induced or service-induced axial stress changes to be evaluated relative to this baseline. The TOF shifts at each loaded stress state were extracted using the procedure described in Section 4.3, and the axial stresses were estimated using the experimental calibration coefficient obtained above. At each stress level, the estimated stress is reported as the mean ± standard deviation of the three repeated acquisitions. The results are summarized in Table 1.

The estimated stresses under all three test conditions showed good agreement with the reference stresses. The mean absolute deviations from the reference stress for Test conditions 1, 2, and 3 were 1.90, 1.64, and 2.05 MPa, respectively, while the corresponding root mean square deviations were 2.01, 1.75, and 2.46 MPa. The mean absolute relative deviations were 3.62%, 2.84%, and 3.06%, respectively. Considering all 12 validation stress states, the overall mean absolute deviation from the reference stress was 1.86 MPa, the root mean square deviation was 2.09 MPa, and the mean absolute relative deviation was 3.17%. The maximum absolute deviation was 4.18 MPa, and the maximum absolute relative deviation was 6.26%.

After probe removal, reinstallation, and recoupling, the mean absolute deviation from the reference stress for Test condition 2 was 1.64 MPa, compared with 1.90 MPa for Test condition 1 before reinstallation. No evident degradation in measurement performance was observed after probe reinstallation and recoupling under the present experimental conditions. To provide a quantitative comparison with the conformal probe, five calibration tests were performed using the conventional planar probe over the same stress range of 0–100 MPa at 20 MPa intervals. Each calibration test used its own zero-load signal as the reference, and the TOF shifts were extracted using the same procedure described in Section 4.3. The five tests yielded a mean TOF stress sensitivity coefficient of 0.8426 ns/MPa, with a standard deviation of 0.0088 ns/MPa and a coefficient of variation of 1.05%. Linear regression of the mean TOF shifts yielded $R^{2}=0.9993$. The mean sensitivity coefficients of the planar and conformal probes differed by approximately 1.5%, indicating comparable stress-induced TOF responses under the tested conditions.

The stress estimation performance of the conventional planar probe was further evaluated under the same three types of validation conditions used for the conformal probe: an initial test, a repeated test after probe removal, reinstallation, and recoupling, and an independent test on another tube specimen. The reference stress levels were 30, 50, 70, and 90 MPa, with three repeated signal acquisitions at each stress level. The fixed mean planar calibration coefficient of 0.8426 ns/MPa was used for all stress estimations without additional correction. Based on the mean estimated stress at each of the 12 validation states, the mean absolute deviation from the reference stress was 4.74 MPa, the root mean square deviation was 5.60 MPa, and the mean absolute relative deviation was 9.20%. The maximum absolute deviation and maximum absolute relative deviation were 10.06 MPa and 25.52%, respectively. For the conformal probe, the corresponding values over the 12 validation states were 1.86 MPa, 2.09 MPa, 3.17%, 4.18 MPa, and 6.26%, respectively.

Table 2 summarizes the quantitative comparison between the conventional planar and conformal probes. The two probe configurations exhibited similar mean TOF stress sensitivities and high calibration linearity. In the validation tests, however, the conformal probe exhibited lower overall stress estimation deviations under the tested conditions. Together with the improved circumferential waveform consistency observed in Section 3.3 and the greater time domain prominence of the target wave packet shown in Figure 8, these results indicate that curvature matching contributes to improving the practical identifiability of the L(0,2)-dominated response and the stability of TOF-based axial stress measurement.

The calibration relationship reported here corresponds to the tube geometry and measurement configuration considered in this study. When the tube diameter or wall thickness changes, the calibration relationship should be re-established. In addition to geometry, ambient temperature variation can influence guided wave velocity and TOF through thermal expansion and temperature-dependent elastic properties [31,32]. All calibration and validation experiments in this study were therefore conducted under controlled laboratory temperature conditions. For applications involving broader temperature variations, temperature-induced TOF changes can be further characterized and incorporated into the calibration framework.

## 5. Conclusions

This study developed an axial stress measurement method for small diameter thin-walled aluminum alloy tubes using an L(0,2)-dominated guided wave response obtained with a conformal oblique incidence probe. Acoustoelastic-guided wave analysis showed that the L(0,2) mode exhibits relatively low dispersion and stable stress sensitivity within the selected operating region. Considering the dispersion characteristics and transient response, 500 kHz was adopted as an appropriate operating frequency for the present sensing configuration. Three-dimensional finite element analysis and circumferential waveform analysis showed that the conformal configuration produced a more regular circumferential wavefield distribution. Together with the SAFE phase-matching condition, group velocity characteristics, and waveform consistency analysis, these results supported the identification of the selected early arriving response as an L(0,2)-dominated wave packet. Experimental comparison showed that the strong subsequent response reached approximately 91% of the target wave packet amplitude for the conventional planar probe, whereas the corresponding ratio was below 31% for the conformal probe, providing a more stable waveform basis for correlation-based TOF estimation. Repeated calibration established an approximately linear relationship between TOF shift and axial tensile stress. Although the two probe configurations exhibited comparable mean TOF stress sensitivities, the mean absolute deviation from the reference stress decreased from 4.74 MPa for the conventional planar probe to 1.86 MPa for the conformal probe, while the mean absolute relative deviation decreased from 9.20% to 3.17%. Validation on different tube specimens, together with repeated measurements after probe reinstallation and recoupling, further demonstrated stable measurement performance under the tested conditions. The results demonstrate the feasibility of quantitative evaluation of axial stress changes relative to a reference state using the proposed conformal guided wave configuration in small diameter thin-walled aluminum alloy tubes under restricted circumferential access. In the present experiments, the zero applied load condition was used as the reference state. For practical applications, a baseline may be established before tube installation under an unloaded and unconstrained condition, enabling subsequent assembly-induced or service-induced axial stress changes to be evaluated relative to this baseline. Future work may incorporate temperature compensation and geometry-dependent calibration to broaden its applicability to more diverse in service conditions.

## Figures and Tables

**Table 1:** Experimental validation results of axial stress measurement.

Test Condition	Reference Stress/MPa	Estimated Stress/MPa	Absolute Deviation/MPa	Absolute Relative Deviation
1	30	31.88 ± 0.65	1.88	6.26%
	50	48.93 ± 0.91	1.07	2.14%
	70	72.91 ± 0.41	2.91	4.16%
	90	88.26 ± 0.39	1.74	1.93%
2	30	29.30 ± 0.44	0.70	2.34%
	50	47.99 ± 1.46	2.01	4.01%
	70	72.36 ± 0.31	2.36	3.37%
	90	91.47 ± 0.39	1.47	1.64%
3	30	29.60 ± 0.80	0.40	1.34%
	50	51.92 ± 0.44	1.92	3.85%
	70	68.32 ± 0.05	1.68	2.40%
	90	94.18 ± 0.66	4.18	4.65%

**Table 2:** Quantitative comparison of the conventional planar and conformal probes.

Metric	Conventional Planar Probe	Conformal Probe
Mean TOF stress sensitivity (ns/MPa)	0.8426	0.8301
CV of sensitivity	1.05%	2.48%
$\mathrm{Calibration}\;R^{2}$	0.9993	0.9994
Mean absolute deviation (MPa)	4.74	1.86
Root mean square deviation (MPa)	5.60	2.09
Mean absolute relative deviation	9.20%	3.17%

## Data Availability

All data supporting the findings of this study are available within the paper.
